# *Streptococcus intermedius* Septic Arthritis of the Acromioclavicular Joint with Periarticular Abscesses in an Elderly Man with Diabetes and Recent Canine Exposure: A Case Report and Literature Review

**DOI:** 10.3390/idr18020021

**Published:** 2026-02-26

**Authors:** Gabriel A. Godart, Vidit Yadav, Elizabeth P. Wellings, Rupert O. Stanborough, Vincent C. Zummo, Bryan D. Springer, Ravi V. Durvasula, Sammer M. Elwasila

**Affiliations:** 1 Division of Infectious Diseases, Department of Medicine, Mayo Clinic, Jacksonville, FL 32224, USA; yadav.vidit@mayo.edu (V.Y.); durvasula.ravi@mayo.edu (R.V.D.); elwasila.sammer@mayo.edu (S.M.E.); 2 Department of Orthopedic Surgery, Mayo Clinic, Jacksonville, FL 32224, USA; wellings.elizabeth@mayo.edu (E.P.W.); zummo.vincent@mayo.edu (V.C.Z.); springer.bryan@mayo.edu (B.D.S.); 3 Department of Radiology, Mayo Clinic, Jacksonville, FL 32224, USA; stanborough.rupert@mayo.edu

**Keywords:** *Streptococcus intermedius*, zoonotic infection, septic arthritis, acromioclavicular joint

## Abstract

Background/Objectives: *Streptococcus intermedius*, a member of the *Streptococcus anginosus* group, is characterized by a marked propensity for abscess formation but only rarely causes native-joint septic arthritis. Involvement of the acromioclavicular (AC) joint is particularly uncommon. We describe a case of native AC joint septic arthritis due to *S. intermedius* in a patient with multiple predisposing factors and highlight diagnostic and management considerations. Methods: We report the clinical course of a 72-year-old man with poorly controlled type 2 diabetes mellitus who presented with progressive right shoulder pain, erythema, and swelling following recurrent minor skin abrasions from a newly adopted dog. Initial management for presumed inflammatory shoulder pathology included brief systemic corticosteroids and an ultrasound-guided intra-articular ketorolac injection. Magnetic resonance imaging (MRI) was performed after symptom progression. The patient underwent operative irrigation and debridement with collection of synovial fluid and deep tissue cultures. Blood cultures and transthoracic echocardiography were obtained to evaluate for systemic involvement. Results: MRI demonstrated multiloculated periarticular abscesses and osteolysis centered on the AC joint. Operative cultures yielded high colony counts of *S. intermedius* from synovial fluid and deep tissues. Blood cultures and echocardiography were negative. The patient required multiple operative debridements with irrigation, adjunctive local antibiotic therapy, and prolonged targeted β-lactam treatment. Clinical and radiographic improvement was achieved following surgical source control and antimicrobial therapy. Conclusions: Native AC joint septic arthritis due to *S. intermedius* is rare. Older age, uncontrolled diabetes, recent intra-articular intervention, and possible zoonotic inoculation from canine wound licking may represent contributory risk factors. Early imaging, prompt surgical source control, and guideline-concordant antimicrobial therapy are essential when bone and soft tissue involvement is present.

## 1. Introduction

The *Streptococcus anginosus* group (SAG) (*S. anginosus, S. constellatus,* and *S. intermedius*) comprises oral commensals notable for pyogenic infections with contiguous spread and multiloculated abscesses. *S. intermedius*, in particular, expresses the human-specific cytolysin intermedilysin, a virulence factor linked to tissue invasion and abscess biology [1,2]. Clinically, SAG predominantly causes head, neck, thoracic, and intra-abdominal abscesses; isolation from native-joint infections is uncommon compared with *Staphylococcus aureus* and other streptococci [3]. Among native-joint infections, acromioclavicular (AC) joint septic arthritis remains rare and often destructive or complicated by adjacent osteomyelitis, mandating early recognition and debridement [4,5]. Regarding antibiotic therapy, the current literature indicates that *S. intermedius* remains largely susceptible to β-lactams, the mainstay of treatment, though emerging resistance to macrolides and clindamycin has been reported [6].

## 2. Microbiological Methods

All specimens were processed using standard aerobic and anaerobic culture techniques. The deep tissue samples were crushed and incubated on standard aerobic (Chocolate agar, aerobic blood agar, Macconkey agar, CNA agar) and anaerobic (LKV Agar, anerobic blood agar, PEA agar) culture media using four-quadrant streaking. Shoulder tissue samples were incubated under strict aerobic and anaerobic conditions, with extended hold times (up to 14 days), to optimize recovery of slow-growing organisms, such as *Cutibacterium acnes*. Species-level identification was performed using matrix-assisted laser desorption/ionization time-of-flight mass spectrometry (MALDI-TOF MS). Susceptibilities were tested using broth microdilution based on CLSI breakpoints.

## 3. Case Presentation

**Figure 1:**
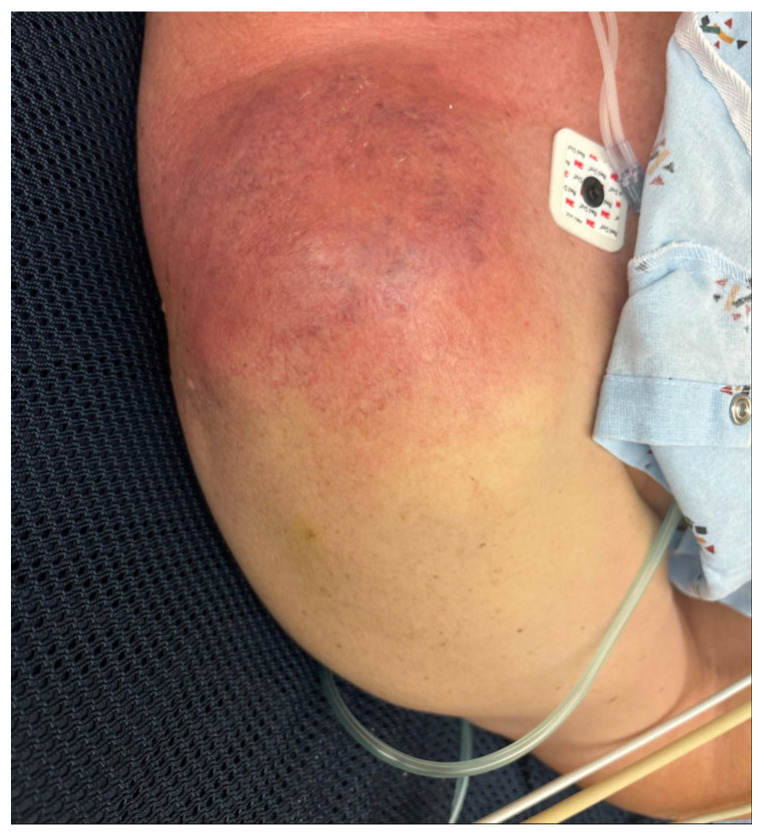
Right shoulder at initial presentation demonstrating marked erythema and edema.

A 72-year-old man with non-insulin-dependent type 2 diabetes mellitus, hypertension, and hyperlipidemia presented to our Emergency Department (ED) in Jacksonville, Florida, with 2 weeks of worsening right-shoulder pain, erythema, and swelling (Figure 1). History revealed recurrent minor scratches on the upper extremities from the patient’s dog, followed by the dog habitually licking these superficial abrasions. He was first evaluated a few days after symptoms arose for presumed superficial infection and was discharged with analgesics, nonsteroidal anti-inflammatory drugs, and 40 mg/day of prednisone for 4 days. One week later, the patient was seen in the orthopedic clinic with concern for adhesive capsulitis; therefore, an intra-articular ketorolac injection was performed. Within 48 h, pain, erythema, and swelling intensified.

**Figure 2:**
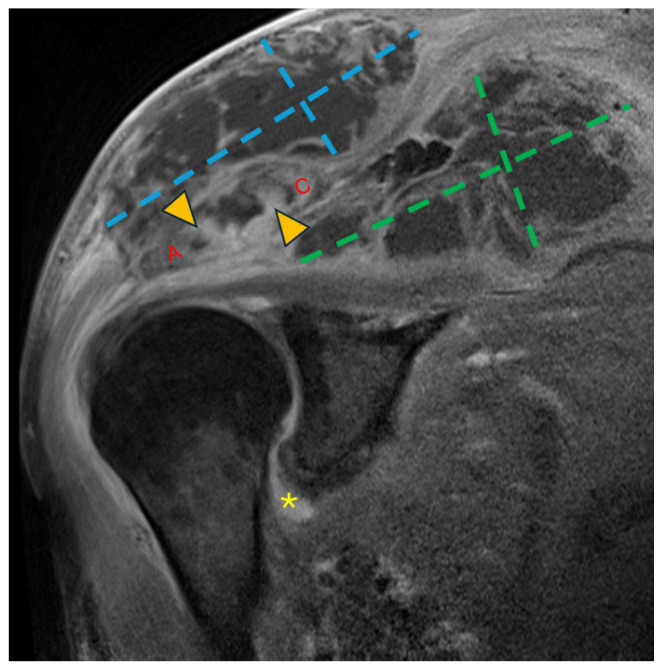
Coronal T1 fat-saturated magnetic resonance image (MRI) post-contrast (gadolinium) at the level of the AC joint (red A = acromion; red C = clavicle). Multilocular peripherally enhancing collections are present in the superficial subcutaneous tissue directly above the AC joint (blue dashed lines) and in the supraspinatus fossa (green dashed lines). Marrow enhancement (yellow arrowheads) of the acromion and clavicle across the AC joint is consistent with osteomyelitis. Synovitis in the axillary recess of the glenohumeral joint (yellow asterisk) raised concern for glenohumeral septic arthritis.

The patient then re-presented to the ED a few days later. He was found to be afebrile and hemodynamically stable. Laboratory studies revealed an elevated white blood cell (WBC) count of 14.0 × 10^9^/L (reference range: 3.4–9.6 × 10^9^/L; all reference ranges below reflect institutional standards), an elevated sedimentation rate (ESR) of 75 mm/h (reference range: 0–22 mm/h), a markedly elevated C-reactive protein (CRP) level of 300.6 mg/L (reference range: <5.0 mg/L), and a recent glycated hemoglobin (HbA1c) of 8.2%. Magnetic resonance imaging (MRI) of the right shoulder demonstrated multiloculated abscesses surrounding the AC joint with associated osteolysis and possible extension into the glenohumeral joint. (Figure 2) Ultrasound-guided arthrocentesis yielded hemorrhagic synovial fluid with a WBC count of 3360/µL with 90% neutrophils. Cultures of the aspirate grew *Streptococcus intermedius* and rare *Cutibacterium acnes.*

He subsequently underwent debridement and irrigation with local antibiotics the following day. Intraoperatively, there was gross purulence within soft tissues anterior and posterior to the AC joint with multiple loculations. The glenohumeral joint was free of gross purulence. Intraoperatively, four deep-tissue samples were obtained. Microscopic examination of the samples revealed many Gram-positive cocci with a few polymorphonuclear and mononuclear cells. Cultures from all four samples grew ‘many’ (4+) colonies of *Streptococcus intermedius* on aerobic blood agar after 2 days; ‘rare’ (1+) *growth of Cutibacterium acnes* was observed in two specimens after 9 days of incubation on anaerobic blood agar. A MALDI-TOF mass spectrometer was used for species-level identification. Given the propensity of *S. intermedius* to form metastatic abscesses, the patient was evaluated for hematogenous dissemination. Blood cultures remained negative; transthoracic echocardiography showed no evidence of infective endocarditis, and the patient exhibited no neurological symptoms or signs suggestive of central nervous system involvement; therefore, CNS imaging was not pursued. Empiric vancomycin (dosed with a goal trough level: 10–15 µg/mL) plus cefazolin (2 g IV q8h) was narrowed to ceftriaxone 2 g IV daily based on susceptibility testing (Table 1) for a planned 6-week course consistent with septic arthritis with adjacent soft-tissue/early osseous involvement, suggestive of developing cellulitis, pyomyositis, and osteomyelitis. At discharge, the WBC count had normalized (7.0 × 10^9^/L) with down-trending ESR (51 mm/h) and CRP (166.3 mg/L).

**Figure 3:**
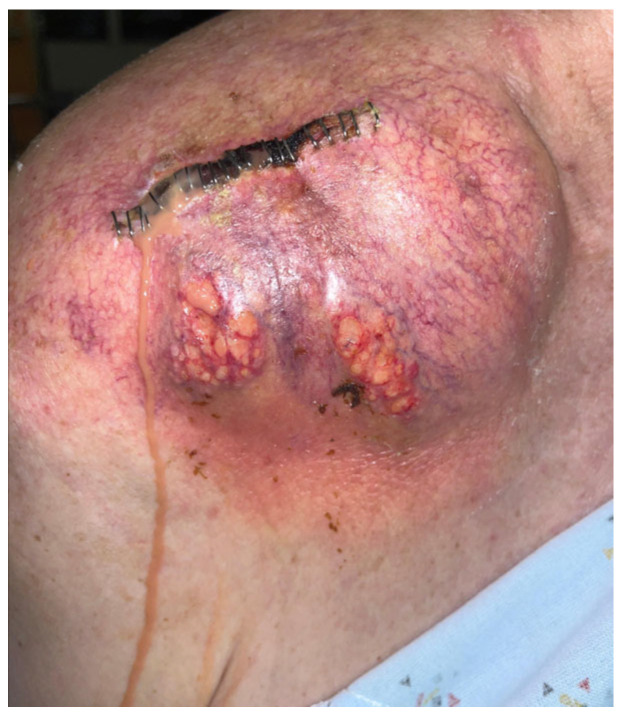
Right shoulder on readmission demonstrating recurrent edema and purulence.

**Figure 4:**
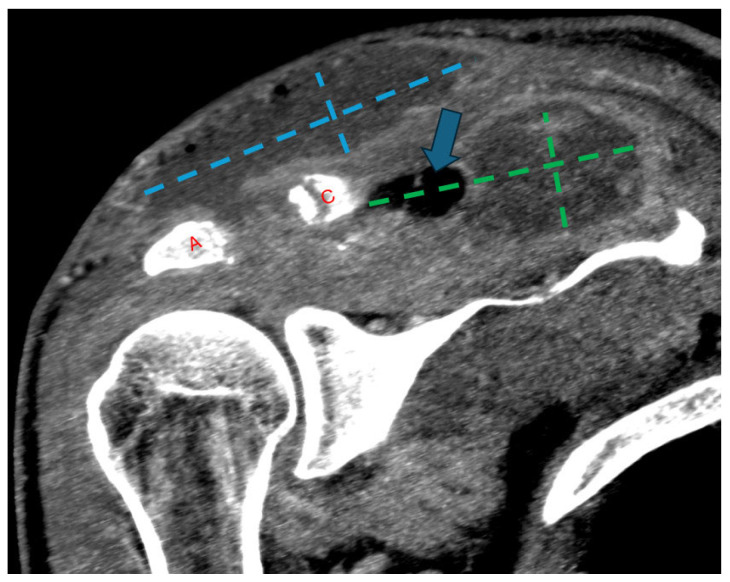
Follow-up coronal computed tomography (CT) image post-contrast at the level of the AC joint (red A = acromion; red C = clavicle). Multilocular peripherally enhancing and gas-containing (blue arrow) collections have redeveloped in the superficial subcutaneous tissue directly above the AC joint (blue dashed lines) and in the supraspinatus fossa (green dashed lines). Progressive widening of the AC joint can be related to interval debridement versus progressive osteomyelitis.

He was readmitted one week later with recurrent edema and purulent drainage (Figure 3) with an elevated WBC count (10.6 × 10^9^/L) and CRP (49 mg/L). Computed tomography (CT) with and without contrast showed extensive multifocal soft-tissue and intramuscular abscesses and progressive osteolysis of the distal clavicle and acromion (Figure 4). The following day, he underwent repeat debridement and irrigation with drain placement. Three deep tissue cultures obtained during repeat operative debridement showed no growth, likely reflecting prior antimicrobial exposure. Despite culture negativity, clinical and radiographic evidence of ongoing infection persisted, including gross purulence at reoperation, extensive multiloculated soft-tissue and intramuscular abscesses on computed tomography, and persistently elevated inflammatory markers requiring repeat surgeries. In this context, antimicrobial therapy was transitioned from ceftriaxone to 2 g of cefazolin IV every 8 h for 6 weeks to optimize musculoskeletal tissue penetration and reduce recurrence risk. At discharge, WBC was 5.7 × 10^9^/L and CRP was 13.2 mg/L with clinical improvement. The patient was recommended home-based physical therapy for reduced range of motion and improved recovery. At 1 month follow-up post completion of antibiotic therapy, the patient was clinically well and stable without any pain, drainage, or systemic symptoms, with normalized inflammatory markers, and no evidence of recurrence.

## 4. Discussion

*S. intermedius* septic arthritis is distinctly uncommon relative to *S. aureus* and other streptococci [3]. When *S. intermedius* does cause invasive disease, it often produces multiloculated abscesses and tissue destruction [1]. The AC joint is an uncommon site of native-joint infection. It is frequently complicated by adjacent osteomyelitis of the acromion or distal clavicle and by periarticular soft-tissue infection, underscoring the value of early cross-sectional imaging and timely surgical management [4,5].

Review of the Literature

To contextualize the rarity and clinical behavior of this organism in native-joint infection, we conducted a focused literature review to identify reported cases of septic arthritis caused by *S*. *intermedius*. Database searches were performed using the terms “*Streptococcus intermedius*” and “*Streptococcus anginosus* group”, combined with “septic arthritis”, “joint infection”, and “septic joint”. Studies were included if they reported species-level identification of *S. intermedius* from synovial fluid, blood, or tissue specimens.

*S. intermedius* is classically associated with intracranial, pulmonary, and intra-abdominal abscesses, whereas musculoskeletal involvement remains rare. To our knowledge, only two published cases of septic arthritis involving native joints have been reported [7,8]. These cases, described by Di Siena A et al. and Yong SM et al., occurred in pediatric patients and involved the hip joint and the atypical atlanto-occipital joint, respectively.

Across reported cases, septic arthritis has been described both in otherwise healthy, immunocompetent individuals and in patients with underlying comorbidities, including rheumatoid arthritis, osteoarthritis, gout, diabetes mellitus, and the presence of prosthetic joints. Reports beyond pediatric cases predominantly involve prosthetic joints or native joints infected by other members of the SAG, such as *S. milleri* or *S. anginosus* [9,10,11,12,13].

Native-joint septic arthritis caused by *S. intermedius* has typically presented as an acute illness characterized by fever, joint pain, and restricted mobility, frequently requiring surgical drainage in addition to intravenous antibiotic therapy [7,8]. Adjacent invasive infections, including deep soft-tissue abscesses and osteomyelitis, have also been reported, underscoring *S. intermedius*’ capacity for both contiguous and hematogenous spread. The majority of reported *S. intermedius* isolates were susceptible to beta-lactam antibiotics and were treated with cephalosporins [7,8,9,10,11,12]. Despite this favorable susceptibility profile and generally low resistance rates, prolonged antimicrobial therapy and surgical intervention are often necessary to achieve adequate source control.

We also performed a focused literature search to identify prior reports of *Streptococcus intermedius* infection associated with canine or zoonotic exposure using the terms “*Streptococcus intermedius*” and “*Streptococcus anginosus* group”, combined with “dog”, “canine”, “zoonotic”, and “animal exposure”. Only human studies published in English were included. Prior human reports have described invasive *S. intermedius* infections temporally associated with close canine exposure, supporting the biological plausibility of dog-associated transmission in susceptible hosts. Subzposh et al. reported an unusual *S. intermedius* infection involving an implantable cardioverter-defibrillator in a dog breeder, highlighting the potential role of repeated canine contact as an epidemiologic risk factor for invasive disease [14]. Similarly, Barnham et al. describe a case of *Streptococcus milleri* septicemia following pig-bite injuries in England [15]. These reports emphasize that, although direct microbiologic confirmation is rarely available, epidemiologic links between animal exposure and invasive *S. intermedius* infection have been documented across diverse clinical settings. Although direct microbiologic confirmation of zoonotic transmission is rarely available in such cases, streptococci, including members of the *Streptococcus anginosus* group, are well-documented components of animal oral flora and are frequently implicated in dog-associated wound infections [16].

Collectively, these reports highlight that *S. intermedius*, although a rare cause of native-joint septic arthritis, should be recognized as a potential pathogen in invasive joint infections, particularly relevant to the present adult AC joint case with suspected zoonotic exposure.

Risk factors

Older age and diabetes mellitus are well-recognized risk factors for native-joint septic arthritis and are common among patients with AC-joint infection [4,17]. The patient’s most recent HbA1c (8.2%) reflected suboptimal glycemic control, which impairs neutrophil function and wound healing [18]. The brief course of systemic corticosteroids, followed by an intra-articular injection for presumed adhesive capsulitis, may have further reduced local host defenses and masked an evolving infection, a scenario noted in reviews of native-joint sepsis and iatrogenic joint infections [17]. Finally, a credible zoonotic pathway exists, as infected dog bite wounds are typically polymicrobial and frequently include streptococcal species, although *Streptococcus intermedius* constitutes a minor proportion relative to other streptococci, such as *S. mitis*, *S. mutans*, and *S. pyogenes*. Importantly, while canine oral flora can plausibly serve as a source for streptococcal inoculation, *Cutibacterium acnes* is not considered a canine-associated organism and is more consistent with human skin flora rather than zoonotic co-transmission [16]. Observational data and reviews document canine oral carriage of human-pathogenic streptococci, and culture-based work has identified *S. intermedius* in canine dental plaque [19]. Given the patient’s recurrent minor scratches and the dog’s repeated licking of superficial abrasions, several biologically plausible pathways exist, including local inoculation of *Streptococcus anginosus* group organisms from upper-extremity skin lesions with contiguous spread to the acromioclavicular region, iatrogenic introduction during the subsequent intra-articular ketorolac injection, or likely hematogenous dissemination [16,19,20].

Diagnostic features

Despite florid periarticular disease, the synovial leukocyte count was modest (3360/µL). Synovial counts < 50,000/µL do not exclude septic arthritis, particularly when infection is predominantly periarticular, when prior host-directed therapies have been given, or when early sampling precedes full intra-articular seeding [21]. Moreover, pre-aspiration antibiotics can depress synovial WBC counts and culture positivity; although this patient had not received antibiotics before aspiration, awareness of this effect helps interpret low counts in similar scenarios [22]. In this case, MRI and CT were decisive in identifying multiloculated abscesses and bone involvement typical of SAG infections and guiding repeated operative source control.

Indications for repeat debridement

Given the multiloculated, tissue-destructive biology of S. intermedius and other SAG infections, and the propensity of acromioclavicular joint sepsis to involve adjacent osteomyelitis, repeat operative debridement is sometimes necessary to achieve durable source control. Reintervention should be considered when clinical improvement is incomplete or transient, inflammatory markers plateau or rise, purulent drainage persists, or cross-sectional imaging demonstrates residual or new collections or progressive osteolysis despite appropriate antimicrobial therapy [1,4,5,17,23]. Because culture yield may diminish after initiation of antibiotics, decisions to reoperate should be guided by the overall clinical course and imaging rather than culture results alone [21,22]. In such settings, staged debridements paired with β-lactam therapy remain consistent with contemporary guidance for native-joint septic arthritis complicated by abscesses and bone involvement [17,23].

Microbiology and treatment

*S. intermedius* typically remains susceptible to β-lactams; rising macrolide resistance has been described [6]. Our use of ceftriaxone, followed by cefazolin, aligns with susceptibility profiles and guideline recommendations for native-joint sepsis, which emphasize de-escalation to culture-directed agents and early surgical drainage when abscesses are present [17,23]. Ceftriaxone and cefazolin offer comparable efficacy and musculoskeletal penetration against *Streptococcus* species, making either suitable for preoperative prophylaxis and the treatment of complicated skin and soft-tissue infections [24]. In patients with a higher body mass index (BMI) or hypoalbuminemia, appropriate weight-based cefazolin dosing and dosing intervals are critical to maintain adequate time above the minimum inhibitory concentration (fT > MIC) in serum and tissues, consistent with the pharmacodynamic properties of β-lactam antibiotics [25,26]. In this context, cefazolin dosed at 2 g intravenously every 8 h aligns with established pharmacokinetic principles for time-dependent β-lactams and is commonly used to ensure adequate tissue exposure in musculoskeletal infections.

*Cutibacterium acnes*

A single, low-burden *C. acnes* isolate from a single specimen in the shoulder region is frequently a contaminant rather than a pathogen; correlation with multiple concordant cultures and a compatible clinical picture is recommended; however, many positive cultures do not indicate true infection [27]. Although *C. acnes* was isolated in low quantity, its role as a true pathogen was considered unlikely. *C. acnes* is a common skin commensal and a frequent contaminant in shoulder cultures, particularly when present in low burden. Given the patient’s clinical trajectory on streptococcal-directed β-lactam therapy without targeted anti-*C. acnes* treatment and the absence of repeated *C. acnes* growth, *S. intermedius* remained the principal pathogen. Although under-detection of *C. acnes* is a possibility, especially with inappropriate anaerobic processing, its rare, non-concordant isolation with extended incubation, and the patient’s clinical response without relapse, argue against a clinically significant role in this case.

Limitations

Although a zoonotic source was considered biologically plausible given repeated canine licking of superficial skin abrasions and known oral carriage of *Streptococcus intermedius* in dogs, direct microbiologic confirmation of zoonotic transmission was not performed. Culturing the dog’s oral flora and performing phenotypic or genotypic comparison (e.g., MLST or PFGE) between human and canine isolates would be required to definitively establish transmission. As such, the zoonotic association in this case remains suggestive rather than proven and is presented to highlight a potential exposure pathway rather than to assert causality. Microbiologic sampling of the dog’s oral flora and screening of the patient for oropharyngeal carriage were not performed, and no active dental infection was identified on clinical assessment.

## 5. Conclusions

Destructive AC-joint septic arthritis due to *S. intermedius* described in this case report illustrates an uncommon manifestation of a classically abscess-forming organism. The patient’s uncontrolled diabetes, recent corticosteroid exposure and intra-articular injection, and dog-related wound contamination constitute a risk profile for invasive SAG infection. Clinicians should consider SAG, particularly *S. intermedius*, when periarticular abscesses and osteolysis are present, especially in the setting of potential oral or animal-saliva exposures. Early imaging as well as prompt and, when necessary, repeated surgical source control, along with approximately six weeks of β-lactam-based therapy when bone is involved, align with current clinical guidelines and antimicrobial susceptibility patterns [6,17,22].

## Figures and Tables

**Table 1:** Antimicrobial Susceptibility of *Streptococcus intermedius* isolated from deep tissue samples. Susceptibility testing on *C. acnes* was not performed due to predictable susceptibility to penicillin. Azithromycin and clarithromycin susceptibility can be inferred from the erythromycin results. MICs are reported in µg/mL, with susceptibility interpreted according to CLSI breakpoints applicable at the time of testing.

Antimicrobial Agent	MIC (µg/mL)	Interpretation
Ceftriaxone	≤0.5	Susceptible
Erythromycin	≤0.25	Susceptible
Levofloxacin	≤2	Susceptible
Penicillin	≤0.06	Susceptible
Vancomycin	≤1	Susceptible

## Data Availability

No new data were created or generated in this case report. All information presented is derived from the patient’s clinical course and existing medical records, and no publicly archived datasets were analyzed. Data sharing does not apply to this article.
